# Niraparib (MK-4827), a novel poly(ADP-Ribose) polymerase inhibitor, radiosensitizes human lung and breast cancer cells

**DOI:** 10.18632/oncotarget.2083

**Published:** 2014-06-09

**Authors:** Kathleen A. Bridges, Carlo Toniatti, Carolyn A. Buser, Huifeng Liu, Thomas A. Buchholz, Raymond E. Meyn

**Affiliations:** ^1^ Department of Experimental Radiation Oncology, The University of Texas MD Anderson Cancer Center, Houston, Texas; ^2^ IRBM/Merck Research Laboratories Rome, Italy; ^3^ Merck Sharp & Dohme Corp., Upper Gwynedd, Pennsylvania; ^4^ Department of Radiation Oncology, The University of Texas MD Anderson Cancer Center, Houston, Texas

**Keywords:** Radiation, PARP, MK-48_2_7, DNA damage, niraparib

## Abstract

The aim of this study was to assess niraparib (MK-4827), a novel poly(ADP-Ribose) polymerase (PARP) inhibitor, for its ability to radiosensitize human tumor cells. Human tumor cells derived from lung, breast and prostate cancers were tested for radiosensitization by niraparib using clonogenic survival assays. Both p53 wild-type and p53-defective lines were included. The ability of niraparib to alter the repair of radiation-induced DNA double strand breaks (DSBs) was determined using detection of γ-H_2_AX foci and RAD51 foci. Clonogenic survival analyses indicated that micromolar concentrations of niraparib radiosensitized tumor cell lines derived from lung, breast, and prostate cancers independently of their p53 status but not cell lines derived from normal tissues. Niraparib also sensitized tumor cells to H_2_O_2_ and converted H_2_O_2_-induced single strand breaks (SSBs) into DSBs during DNA replication. These results indicate that human tumor cells are significantly radiosensitized by the potent and selective PARP-1 inhibitor, niraparib, in the *in vitro* setting. The mechanism of this effect appears to involve a conversion of sublethal SSBs into lethal DSBs during DNA replication due to the inhibition of base excision repair by the drug. Taken together, our findings strongly support the clinical evaluation of niraparib in combination with radiation.

## INTRODUCTION

There is increasing interest in combining molecularly targeted agents with conventional therapeutics, including chemotherapy drugs and ionizing radiation, for the treatment of human cancer [1]. Based on the fact that the cytotoxicity of radiation and many such drugs is due to the induction of damage to DNA, a promising strategy for enhancing the effectiveness of these treatments is to inhibit the pathways for repairing these DNA lesions [2]. Thus, there has been a concerted effort to develop molecularly targeted agents that specifically inhibit DNA repair processes. In the case of ionizing radiation, the principal DNA lesions are single strand breaks (SSBs) and double strand breaks (DSBs) [3]. Although DSBs are considered to be the main lethal lesions [4], SSBs can also contribute to lethality if their repair is compromised thereby causing them to be converted to DSBs. SSBs are repaired by the base excision repair (BER) pathway [5] and DSBs are repaired through 2 pathways, the non-homologous end joining (NHEJ) and the homologous recombination repair (HRR) pathways [4, 6].

These DNA repair pathways are generally very complex and are comprised of the activities of many different proteins. Thus, many targets can be identified for inhibition that would suppress DNA repair and lead to the increased cytotoxicity of DNA-damaging chemotherapy drugs and radiation. However, in spite of this abundance of potential targets, one DNA repair protein in particular, poly(ADP-ribose) polymerase 1 (PARP-1), has received considerable attention. Several pharmaceutical companies have developed novel PARP inhibitors and initiated clinical trials with these agents either alone or in combination with DNA-damaging drugs such as cisplatin [7-9]. PARP-1 is a member of a family of 18 such proteins of which only PARP-1 and 2 are known to bind to DNA and function in DNA repair [10, 11]. Essential to its activity, PARP-1 has an N-terminal DNA binding domain that enables it to bind to SSBs and DSBs [12]. Following DNA binding, PARP-1's enzymatic activity is triggered and it functions to add poly(ADP-ribose) polymers to histones and other proteins including itself [10]. This in turn facilitates the recruitment of DNA repair proteins to the site of the DNA lesions. PARP-1 is essential for the repair of SSBs by the BER pathway where it enables the recruitment of proteins key to BER such as DNA polymerase β and XRCC1 [13, 14]. Thus, inhibition of PARP-1 can sensitize tumor cells to classes of DNA damaging drugs that induce lesions subject to the BER repair pathway such as temozolomide, cyclophosphamide, camptothecin, etc. [15-17]. PARP-1 is also known to function in DSB repair where it mediates the recruitment of MRE11 and NBS, proteins key to the DSB repair pathways, NHEJ and HRR [18]. Therefore, PARP-1 inhibitors potentially radiosensitize tumor cells via 2 independent mechanisms; 1) inhibition of SSB repair could lead to the ultimate conversion of SSBs to DSBs when DNA replication is attempted past unrepaired SSBs during S phase and 2) the prolongation of unrepaired DSBs when DSB repair is inhibited.

The important role of PARP in radioresponse and the efficacy of PARP inhibitors as radiosensitizers have been investigated for more than 30 years. Indeed, the depletion of cellular NAD, the substrate for ADP-ribose polymers, in irradiated cells and whole animals was initially demonstrated in the 1950s before the enzyme PARP was even discovered in the early 1960s [19]. The first known PARP inhibitors, nicotinamide and 3-aminobenzamide (3-AB) and their derivatives, were subsequently shown to suppress NAD depletion in irradiated cells leading to an enhancement of the cytotoxic effects of radiation and this earlier work has been reviewed previously [19, 20]. Zwelling et al. used the technique of alkaline elution to show that 3-AB suppressed the repair of radiation-induced SSBs [21] and our laboratory used this method to show repair of H_2_O_2_-induced SSBs were repaired more slowly in the presence of 3-AB correlating with an enhanced H_2_O_2_ cytotoxicity by 3-AB [22].

Due to its lack of potency and specificity, 3-AB is not clinically useful. Therefore, a number of third-generation PARP inhibitors, some derived from the 3-AB structure, have been developed in recent years and tested in pre-clinical and clinical studies including olaparib from AstraZeneca, veliparib (ABT-888) from Abbot, INO-1001 from Inotek, AG014699 from Pfizer, and niraparib (formally known as MK-4827) from Merck [23]. All of these agents are in phase I or phase II clinical trials for various solid tumors either alone or in combination with conventional chemotherapy drugs such as cisplatin [7, 8]. Olaparib [24], INO-1001 [25], ABT-888 [25, 26], and AG014361 [15] have all been shown previously in preclinical investigations to radiosensitize various human cell lines in vitro. Here, we report the results of our tests of the PARP-1 inhibitor, niraparib, for its ability to radiosensitize non-small cell lung cancer (NSCLC), breast cancer and prostate cancer cell lines treated in vitro. Our results demonstrate that niraparib has potent radiosensitizing properties that correlate with its ability to inhibit DNA repair processes.

## RESULTS

### Niraparib radiosensitizes human tumor cells in a p53-independent manner

**Fig 1:**
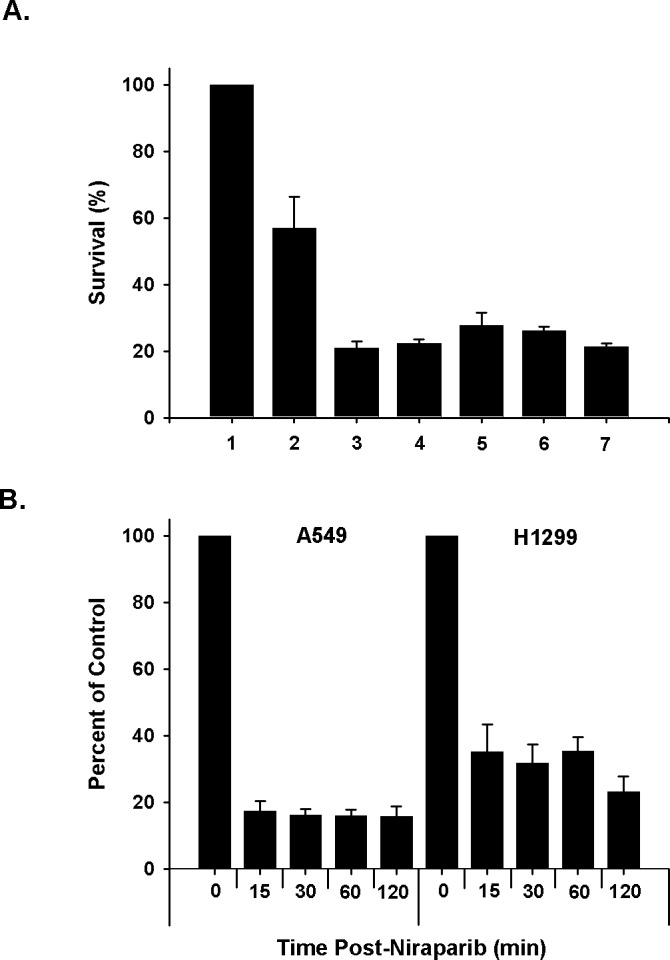
Test of optimal *in vitro* sequencing of niraparib and radiation and time course of inhibition A. A549 cells were treated with different sequencing protocols combining 1 μmol/L of niraparib (MK) and 4 Gy of radiation. Following the treatments, the cells were plated for clonogenic survival. 1. unirradiated control; _2_. 4 Gy; 3. MK (1 h) + 4 Gy + MK (_2_4 h); 4. MK (1 h) + 4 Gy + MK (1 h) + no drug (_2_3 h); 5. MK (1 h) + 4 Gy + no drug (_2_4 h); 6. 4 Gy + no drug (1 h) + MK (_2_3 h); 7. 4 Gy + MK (_2_4 h). B. A549 and H1_2_99 cells were treated with 1 μmol/L of MK and samples were collected as a function of time and analyzed for PARP inhibition using the HT Universal Chemiluminescent PARP Assay Kit. The results shown represent the average of 3 or more independent determinations. Error bars represent the standard error.

We assessed the ability of niraparib to radiosensitize human tumor cells using clonogenic survival curve assays. Various cell lines were tested including lines derived from non-small cell lung cancer (NSCLC), breast cancer, and prostate cancer. The p53 status of all of these lines had been previously established. The optimal concentration of niraparib and sequence of administration relative to irradiation were validated in preliminary, pilot studies using the NSCLC lines A549 and H1299. We determined that 24 h treatments with 1 μmol/L prior to irradiation were only modestly effective. In a detailed test of various treatment sequences using A549 cells, we found that the optimum sequence consisted of a 1 h pre-treatment followed by a 24 h post-irradiation treatment (Fig. 1A) and this treatment strategy was used unless otherwise noted. To validate that niraparib inhibited PARP in these cell lines, we treated A549 and H1299 cells with 1 μmol/L niraparib for various times and measured PARP enzymatic activity using a chemiluminescent assay. The results shown in Fig. 1B show that this concentration of niraparib inhibits PARP within 15 minutes of treatment reaching about 85% inhibition in the A549 cells at 1 h and about 55% inhibition at 1 h for the H1299 cells.

**Fig 2:**
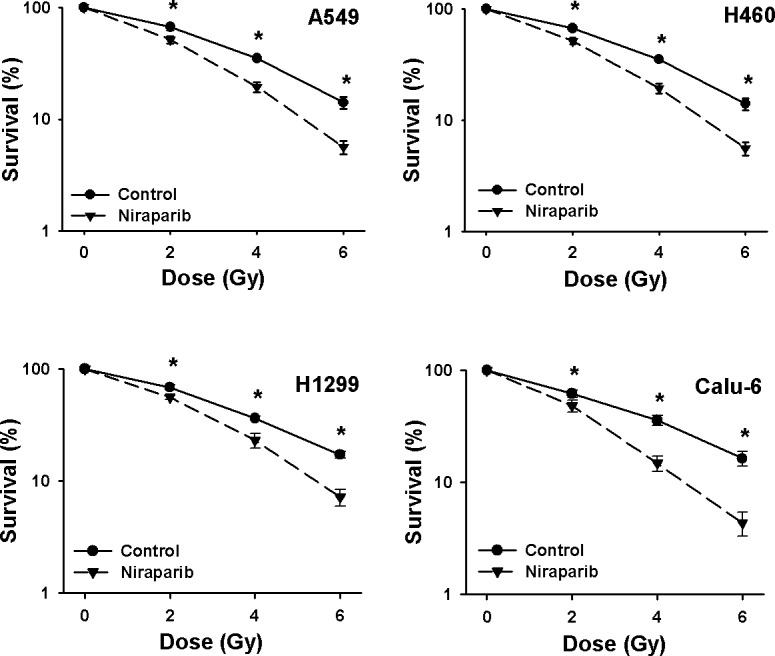
Niraparib radiosensitizes NSCLC cells in a p53-independent manner Clonogenic survival curves for A549 and H460 (both p53 wild-type) and H1299 and Calu-6 (both p53-defective) cells were treated or not with 1 μmol/L of niraparib for 1 h prior to irradiation followed by an additional 24 h post-irradiation incubation in niraparib containing medium. The results shown represent the average of 3 or more independent determinations. Error bars are shown when larger than the symbol plotted and represent the standard error. * indicates *p*<0.05.

**Fig 3:**
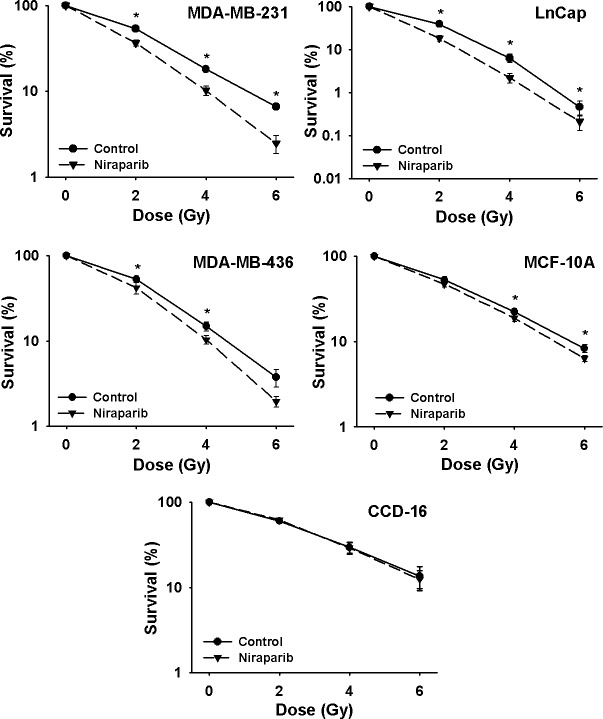
Niraparib radiosensitizes human prostate and breast cancer cells but does not radiosensitize human cells derived from normal tissues Clonogenic survival curves for MDA-MB-231, LnCaP, MDA-MB-436, CCD-16, and MCF-10A cells were treated or not with 1 μmol/L of niraparib for 1 h prior to irradiation followed by an additional 24 h post-irradiation incubation in niraparib containing medium. The results shown represent the average of 3 or more independent determinations. Error bars are shown when larger than the symbol plotted and represent the standard error. * indicates *p*<0.05.

Complete clonogenic survival curves for the 4 NSCLC lines examined consisting of two with wild-type p53, A549 and H460, and two that are null for p53, H1299 and Calu-6, were generated (Fig. 2). A549, H1299, Calu-6 and H460 cells were radiosensitized independently of their p53 status. The breast cancer lines and the prostate cancer line were also effectively radiosensitized by niraparib (Fig. 3). The degree of radiosensitization was quantified from the survival curves in two different ways; by comparing the surviving fractions at the radiation doses of 2 and 4 Gy (SF2 and SF4) and by calculating the dose enhancement factor (DEF), i.e. the ratio of radiation doses to achieve a given survival level. The DEF values for all of the cell lines examined are provided in Table 1. All of the tumor cell lines examined displayed some degree of radiosensitization by niraparib independently of their p53 status and many had substantial and significant changes in SF2 and SF4 values. For example, for A549 cells, SF4 was reduced from 0.35 ± 0.07% in the control to 0.19 ± 0.03% (p=0.002) by niraparib.

**Table 1:** The DEF values calculated from the survival curves shown in Figures 2 and 3 for the p53 defective and p53 wild-type cell lines

p53 wild-type cell lines	DEF	p53 defective cell lines	DEF
A549	1.32	H1299	1.34
H460	1.42	MDA-MB-231	1.36
MCF-10A	1.10	Calu-6	1.61
LnCap	1.43	MDA-MB-436	1.25
CCD-16	1.00		

Niraparib at the concentration of 1 μmol/L was slightly toxic to these cell lines, typically lowering PE by about 20%. For example, for the two cell lines used in subsequent experiments, the PE for A549 cells was reduced from 80.3 ± 3.8% in controls to 61.2 ± 14.3% in niraparib treated cells and in H1299 cells PE was reduced from 86.5 ± 2.3% to 65.8 ± 11% by niraparib. These effects were typical for the other tumor cell lines tested, independently of their p53 status and none of these reductions in PE reached statistical significance with the exception of the BRCA1-defective cell line, MDA-MB-436 [27], where PE was reduced by niraparib from 11.5 ± 1.1% to 2.5 ± 0.9% (p=0.004). Additionally, for the two cell lines derived from normal tissues, CCD-16 (normal lung fibroblasts) and MCF-10A (normal breast epithelial cells); PE was not affected by niraparib in these cell lines. Moreover, these two lines were only slightly radiosensitized by niraparib in the case of the MCF-10A line or not radiosensitized in the case of the CCD-16 cells (Fig. 3 and Table 1).

### Niraparib alters the kinetics of radiation-induced DSBs and their repair

**Fig 4:**
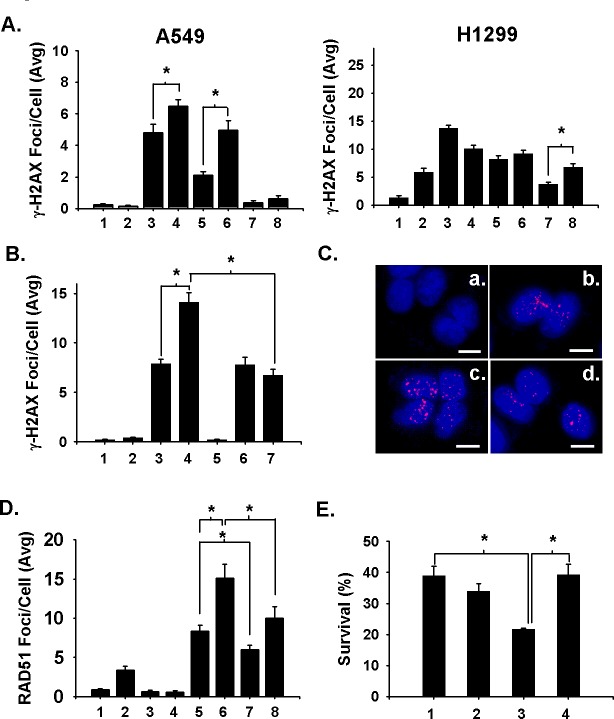
Niraparib enhances the presence of radiation-induced DSBs in A549 and H1299 cells by converting SSBs to DSBs during DNA replication A. A549 and H1299 cells were treated or not with 1 μmol/L of niraparib (MK) for 1 h prior to irradiation with 2 Gy. Samples were then incubated for various times after irradiation and analyzed for DSBs on the basis of γ-H2AX foci as detected by immunofluorescence. 1. Untreated control; 2. MK for 24 h; 3. 2 Gy alone analyzed 1 h after irradiation; 4. MK for 1 h prior to 2 Gy irradiation and analyzed 1 h after irradiation; 5, 2 Gy analyzed 4 h after irradiation; 6. MK for 1 h prior to 2 Gy irradiation and analyzed 4 h after irradiation; 7. 2 Gy analyzed 24 h after irradiation; 8. MK for 1 h prior to 2 Gy irradiation and analyzed 24 h after irradiation. MK was present during the post-irradiation incubations where indicated. B. Aphidicolin suppresses niraparib's ability to enhance radiation-induced DSBs. A549 cells were treated or not with 1 μmol/L of MK for 1 h prior to irradiation with 2 Gy. Samples were then incubated for various times after irradiation and analyzed for DSBs on the basis of γ-H2AX foci as detected by immunofluorescence. Aphidicolin (2 μmol/L) was added to some samples following irradiation to suppress the conversion of SSBs to DSBs during DNA replication. 1. Untreated control; 2. MK alone; 3. 2 Gy alone analyzed 1 h after irradiation; 4. MK for 1 h prior to irradiation and analyzed 1 h after irradiation; 5. aphidicolin alone; 6. 2 Gy analyzed 1 h after irradiation plus aphidicolin; 7. MK for 1 h prior to irradiation and analyzed 1 h after irradiation plus aphidicolin. MK was present during the post-irradiation incubations where indicated. C. Representative photomicrographs of γ-H2AX foci for key samples for Fig. 4B. a. unirradated control; b. 2 Gy; c. 2 Gy + MK; d. 2 Gy + MK + aphidicolon. Bar is 10 microns. D. Aphidicolin suppresses niraparib's ability to enhance radiation-induced DSBs detected as RAD51 foci. A549 cells were treated or not with 1 μmol/L of MK for 1 h prior to irradiation with 5 Gy. Samples were then incubated for various times after irradiation and analyzed for DSBs on the basis of RAD51 foci as detected by immunofluorescence. Aphidicolin (2 μmol/L) was added to some samples following irradiation to suppress the conversion of SSBs to DSBs during DNA replication. 1. Untreated control; 2. MK alone; 3. Aphidicolin alone; 4. MK + aphidicolin; 5. 5 Gy alone analyzed 4 h after irradiation; 6. MK for 1 h prior to irradiation and analyzed 4 h after irradiation; 7. 5 Gy analyzed 4 h after irradiation plus aphidicolin; 8. MK for 1 h prior to irradiation and analyzed 4 h after irradiation plus aphidicolin. MK was present during the post-irradiation incubations where indicated. E. Aphidicolin suppresses niraparib's radiosensitization of clonogenic survival. A549 cells were treated with 1 μmol/L of niraparib (MK) for 1 h prior to irradiation with 4 Gy. Aphidicolin was added to some samples following irradiation for 2 h prior to plating for clonogenic survival. 1. 4 Gy alone; 2. 4 Gy plus aphidicolin; 3. MK for 1 h prior to and 2 h post-irradiation; 4. MK for 1 h prior to 4 Gy plus aphidicolin. The results shown represent the average of 3 or more independent determinations. Error bars are shown when larger than the symbol plotted and represent the standard error. * indicates p<0.05.

Radiation induces both SSBs and DSBs but the DSBs are considered the principal lethal lesions [4]. Thus, we tested whether the radiosensitization of NSCLC cells by niraparib involved an alteration in the repair of radiation-induced DSBs. A549 and H1299 cells were pre-treated or not for 1 h with niraparib and then irradiated with 2 Gy. Samples were collected as a function of time following irradiation and analyzed for the presence of γ-H2AX foci to detect DSBs. The results, shown in Fig. 4A, indicate that radiation-induced DSBs were prolonged by niraparib in A549 and H1299 cells as a function of time after treatment generally correlating with the ability of niraparib to radiosensitize these lines (Fig. 1). The kinetics of this effect were different in the 2 cell lines where the enhancement was evident at early times after irradiation for the A549 cells and for later times for the H1299 cells. It was not possible from this experiment to distinguish whether niraparib enhanced DSBs by altering, following irradiation, their further production or by suppressing their repair.

Although PARP inhibitors can inhibit DSB repair, they can also induce the conversion of SSBs to DSBs during DNA replication by inhibiting BER. To ascertain the possible contribution of this later mechanism, we repeated the assessment of γ-H2AX foci in irradiated A549 cells in the presence or absence of 2 μmol/L aphidicolin to inhibit DNA replication. A549 cells were treated for 1 h with niraparib or not and then irradiated with 2 Gy. Cells were harvested after 1 h of additional incubation with or without niraparib. Aphidicolin was added or not during the 1-h post-irradiation incubation period. The results show that niraparib treatment alone enhances DSBs detected as γ-H2AX foci 1 h after irradiation (Fig. 4B) similar to what was seen before (Fig. 4A). However, when aphidicolin was added during the 1-h post-irradiation incubation period, the ability of niraparib to enhance radiation-induced DSBs was suppressed to levels comparable to radiation alone suggesting that the enhancement of radiation-induced DSBs by niraparib is primarily due to a conversion of SSBs to DSBs during DNA replication through its ability to inhibit BER. Representative photomicrographs illustrating γ-H2AX foci for some of these treatments are presented in Figure 4C. We also examined the induction of RAD51 foci in an experiment comparable to that for the γ-H2AX foci. RAD51 has been identified as a marker for DSBs undergoing HRR and, specifically, for HRR-mediated repair of DSBs resulting from replication forks stalled at unrepaired SSBs [4, 28, 29]. The results (Fig. 4D) show that radiation alone induces some RAD51 foci and that these are enhanced by niraparib. Aphidicolin substantially suppressed the ability of niraparib to enhance radiation-induced RAD51 foci. It also suppressed RAD51 foci levels induced by radiation alone suggesting that a small proportion of DSBs induced by radiation result from the conversion of SSBs to DSBs at stalled replication forks. Representative photomicrographs illustrating RAD51 foci for some of these treatments are presented in Figure S1. We also assessed whether aphidicolin altered niraparib's radiosensitization of clonogenic survival. The results shown in Fig. 4E show that while aphidicolin did not alter the radiosensitivity of A549 cells in the absence of niraparib treatment, it did completely abrogate the radiosensitization effect of niraparib.

### Niraparib converts H_2_O_2_-induced SSBs to DSBs during DNA replication

**Fig 5:**
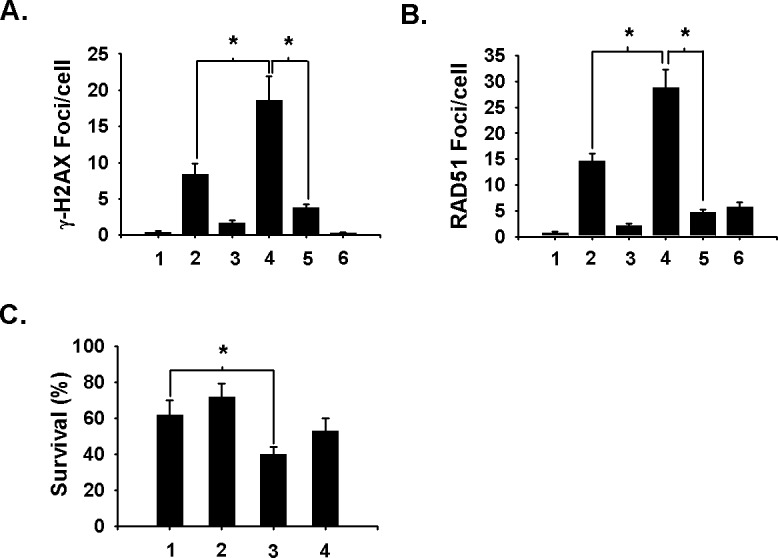
Aphidicolin suppresses niraparib's ability to enhance HO-induced DSBs A549 cells were treated or not with 1 μmol/L of niraparib (MK) for 1 h prior to treatment with 200 μmol/L H_2_O_2_ for 15 min. Samples were then incubated for 30 minutes after irradiation and analyzed for DSBs on the basis of γ-H2AX (A) or RAD51 (B) foci as detected by immunofluorescence. Aphidicolin (2 μmol/L) was added to some samples before and after H_2_O_2_ treatment to suppress the conversion of SSBs to DSBs during DNA replication. 1. Untreated control; 2. H_2_O_2_ alone; 3. MK alone; 4. MK for 1 h prior to H_2_O_2_ and analyzed 30 min after treatment; 5. MK and aphidicolin for 1 h prior to H_2_O_2_ and analyzed 30 min after H_2_O_2_; 6. Aphidicolin for 1 h prior to H_2_O_2_ and analyzed 30 min after treatment. MK and aphidicolin were present during the post-H_2_O_2_ incubations where indicated. C. Aphidicolin suppresses niraparib's sensitization of H_2_O_2_ toxicity assessed using clonogenic survival. A549 cells were treated with 1 μmol/L of niraparib for 1 h prior to treatment with 100 μmol/L H_2_O_2_ for 15 min. Aphidicolin was added to some samples for a 1 h pre-treatment and for 30 minutes following H_2_O_2_ treatment prior to plating for clonogenic survival. 1. H_2_O_2_ alone; 2. H_2_O_2_ plus aphidicolin; 3. MK for 1 h prior to H_2_O_2_; 4. MK and aphidicolin for 1 h prior to and 30 min after H_2_O_2_. The results shown represent the average of 3 or more independent determinations. Error bars represent the standard error. * indicates *p*<0.05.

To further test niraparib's ability to inhibit BER and convert SSBs to DSBs during DNA replication, we used H_2_O_2_ to induce essentially pure SSBs. Thus, A549 cells were treated with niraparib or not for 1 h and then exposed to H_2_O_2_ for 15 min at 37 degrees. Following H_2_O_2_ treatment, cells were incubated for 30 min in the presence or absence of aphidicolin to inhibit DNA replication and analyzed for the formation of DSBs on the basis of γ-H2AX foci. The results are shown in Fig. 5A where it can be seen that whereas H_2_O_2_ is capable of inducing some foci, niraparib sensitizes A549 cells to H_2_O_2_-induced γ-H2AX foci. Aphidicolin abrogated the ability of H2O2 alone to induce γ-H2AX foci and greatly suppressed the ability of niraparib to enhance H_2_O_2_-induced γ-H2AX foci suggesting that H_2_O_2_ does induce lesions consistent with DSBs due to some conversion of SSBs to DSBs during DNA replication. Inhibition of BER by niraparib augments this effect as expected and aphidicolin blocks this effect. We also examined the induction of RAD51 foci in a comparable experiment. As mentioned above, RAD51 has been identified as a marker for DSBs undergoing HRR and, thus, we also examined the induction of RAD51 foci by H_2_O_2_. The results (Fig. 5B) show that H_2_O_2_ alone induces some RAD51 foci and that these are enhanced by niraparib. Aphidicolin substantially suppressed the ability of niraparib to enhance H_2_O_2_-induced RAD51 foci. It also suppressed RAD51 foci levels induced by H_2_O_2_ alone suggesting that most DSBs induced by H_2_O_2_ alone are also resulting from the conversion of SSBs to DSBs at stalled replication forks. We also tested whether niraparib sensitized H_2_O_2_'s cytotoxicity assessed on the basis of clonogenic survival. The results shown in Fig. 5C show that niraparib sensitizes A549 cells to the cytotoxic effects of a mild H_2_O_2_ treatment. Aphidicolin suppressed to some extent niraparib's sensitization but this effect did not reach statistical significance suggesting that H_2_O_2_ cytotoxicity is not completely mediated by conversion of SSBs to DSBs during DNA replication but may involve other aspects of SSB repair or toxic effects of H_2_O_2_ that don't involve DNA damage.

## DISCUSSION

There is increasing interest in PARPi as therapeutic agents for the treatment of cancer [23]. Several such drugs have been developed in recent years and subjected to preclinical and clinical evaluation [30]. In this study, we investigated the radiosensitizing abilities of niraparib, a novel inhibitor of PARP-1/2. Niraparib may be an especially potent PARPi. In a side-by-side comparison with olaparib and veliparib, Murai et al. [31] showed that niraparib was the most potent of these inhibitors for trapping the PARP-DNA complexes that contribute to PARPi cytotoxicity. They further reported that, based on this mechanism, niraparib displayed the most cytotoxicity to BRCA2-defective cells of the three PARPi tested.

We focused our tests of niraparib on cell lines derived from three types of human tumors, i.e. NSCLC, breast and prostate, where radiotherapy typically plays a key role in the management of patients with these tumors and where improvements in radioresponse in these disease sites would be expected to provide clinical benefit. As shown in Figures 2 and 3 and summarized in Table 1, four p53-defective human tumor cell lines and three tumor cell lines with wild-type p53 were all radiosensitized by niraparib. This comparison of p53-defective and p53 wild-type cell lines suggests that the radiosensitizing effect of niraparib is independent of their p53-status.

Cell lines of normal tissue origin were not sensitized or sensitized to a lesser extent than were the tumor cell lines and this correlated with the lack of a cytotoxic effect of niraparib on these normal cells. As mentioned above, niraparib was mildly cytotoxic to all of the tumor cell lines reducing PE by about 20% whereas the normal cells did not display any toxic effects. This suggests that the DNA repair systems targeted by niraparib are not robustly expressed in the normal cells compared to tumor cells. Indeed, at least in the case of the lung lines, the normal lung fibroblast line, CCD-16, was more radiosensitive compared to the four NSCLC lines. The four NSCLC lines, when taken together, had an average SF2 of 0.69 ± 0.025 whereas the CCD-16 line had an SF2 of 0.59 ± 0.025 and this difference was statistically significant, p=.006. Gorgoullis et al. [32] reported that, in the case of the human lung, precancerous lesions and NSCLC tumors display spontaneous DSBs compared to the normal lung tissue presumably due to oncogene-induced replicative stress suggesting that the DSB repair systems are already activated in tumor cells compared to normal cells. The lack of radiosensitization of the normal cell lines by niraparib would be consistent with that hypothesis.

Although the radiosensitizing effect of inhibiting PARP was first demonstrated more than 30 years ago, the resurgence of interest in PARPi for sensitizing tumor cells to radiation has been generated by the development of third-generation PAPRi over the past decade that may have clinical utility. Several of these newer PARPi have been previously examined for their radiosensitizing properties. Of these, ABT-888 (veliparib) has been tested to the greatest extent. Albert et al., showed that ABT-888 reduced clonogenic survival in irradiated NSCLC cells consistent with an inhibition of radiation-induced DNA damage [26]. ABT-888's radiosensitizing effects have since been confirmed for other NSCLC lines as well as for prostate cancer cells, glioblastoma, cervical cancer cells and colorectal cancer cells [33-37]. Another PARPi, AZD2281 (olaparib), has been reported to increase the radiosensitivity of NSCLC cells consistent with an increased persistence of radiation-induced DSBs [24]. Two additional PARPi, E7016 [38] and INO-1001 [25], have both been shown to enhance the radiosensitivity of various cell lines. Finally, AG14361 has been shown to radiosensitize colorectal cancer cells [15] and, in a recent report, niraparib was shown to radiosensitize human neuroblastoma cells [39]. Additional reports illustrating the radiosensitizing effects of these and other PARPi have been recently reviewed [40].

Ionizing radiation induces various lesions in DNA including DSBs, base damage and SSBs [3]. DSBs are repaired by NHEJ and HRR [4, 6]. Base damage and SSBs are repaired by BER [5]. DSBs represent the lethal lesions induced by radiation because they are difficult to repair with fidelity whereas base damage and SSBs do not normally contribute to lethality due to the very efficient and accurate BER repair process [4]. However, SSBs can contribute to lethality if they are converted to DSBs through an inhibition of BER [40]. PARPi, through their ability to inhibit BER, cause some SSBs to be processed into DSBs when DNA replication encounters an unrepaired lesion [9, 41]. We observed that niraparib appeared to radiosensitize by prolonging the presence of DSBs in irradiated cells based on the detection of γ-H2AX foci (Fig. 4A). However, this analysis could not distinguish between an inhibition of repair of DSBs by niraparib versus a conversion of SSBs to DSBs by the drug. Thus, in analogy with previous publications [29, 42], we used aphidicolin to block DNA replication and tested whether this would affect the level of γ-H2AX foci produced by niraparib in irradiated cells. The results (Fig. 4B) indicated that aphidicolin lowered DSBs in niraparib-treated cells to control levels seen with radiation alone suggesting that the additional DSBs induced by niraparib in irradiated cells were due to the conversion of SSBs to DSBs during S phase.

We also observed that the kinetics of DSB prolongation by niraparib were different between the A549 and H1299 cells (Fig. 4A). This could be due to the different p53 status of these 2 cell lines; A549 cells having wild-type p53 and H1299 cells having null p53 status. As mentioned above, niraparib would be expected to primarily enhance DSBs repaired through the HRR pathway which has been shown to preferentially occur during late S/G2 [43]. Cells with wild-type p53 have 2 independent mechanisms to accumulate irradiated cells in G2 phase thereby allowing additional time for repairing DSBs prior to entering mitosis whereas cells with defective p53 totally rely on activation of wee1 kinase [44]. In a previous report from our laboratory [45], we showed that during the first 4 h following irradiation, A549 cells accumulate in G2 more than twice as fast as H1299 cells. Thus, the difference in kinetics of DSB prolongation seen between these 2 cell lines (Fig. 4A) may be due to this difference in the rate of progression through late S and into G2 phase, the portion of the cell cycle where niraparib would maximally exert its enhancement of DSBs.

We conducted further experiments using H_2_O_2_ to confirm that niraparib acts to convert SSBs to DSBs. Although H_2_O_2_ can produce an occasional DSB due to closely spaced SSBs on opposite DNA strands, it essentially induces a relatively clean spectrum of SSBs when used at physiological concentrations [22]. The ratio of SSBs to DSBs has been estimated to be about 18:1 for ionizing radiation and about 3200:1 for H_2_O_2_ [46]. H_2_O_2_ alone induced some detectable γ-H2AX foci but niraparib enhanced the production of these as shown by our results (Fig. 5A). Apidicolin reversed this effect of niraparib similar to what was seen with radiation. In addition to using γ-H2AX foci to indicate the DSBs produced under these conditions, we also examined the presence of RAD51 foci in these studies using H_2_O_2_. It has been shown previously that the DSBs resulting from stalled replication forks are repaired by HRR and that RAD51 is a specific marker for lesions undergoing repair through this pathway [28, 29, 42]. This analysis confirmed that niraparib initiated DNA lesions in H_2_O_2_-treated cells subject to HRR but these were abrogated by aphidicolin (Fig. 5B). These findings with H_2_O_2_ further indicate that niraparib acts similar to previously investigated PARPi with regard to the mechanism of radiosensitization involving the conversion of SSBs to DSBs at stalled DNA replication forks.

Many of the PARPi currently under evaluation have been shown previously to have preferential cytotoxic activity for tumor cells with mutated or non-functional BRCA1 or BRCA2 [7, 23, 47-49]. In the original report of the discovery of niraparib, its preferential cytotoxic effect for BRCA1 defective cells was demonstrated using HeLa cells in which BRCA1 had been silenced [27]. Here we validated that finding using the MDA-MB-436 cells which have mutated BRCA1. Niraparib had substantially greater cytotoxic effect on the MDA-MB-436 cells lowering plating efficiency by about 80% compared to the other cell lines used in our study where niraparib alone lowered plating efficiency by only about 20%. Also, MDA-MB-436 cells appeared to be slightly more radiosensitive than the MDA-MB-231 cells that have wild-type BRCA1 and this is the expected result considering that BRCA1 is an important component of HRR repair [2, 18]. The fact that MDA-MB-436 cells were radiosensitized by niraparib (Fig. 3) suggests that radiotherapy combined with PARPi such as niraparib has efficacy even for tumors with mutant BRCA1.

Although the present report concerns the radiosensitizing effects of niraparib in vitro, niraparib has been previously tested by other members of our team for its ability to sensitize in vivo using xenografts made from four of the cell lines used in this report, A549, H460, MDA-MB-231 and Calu-6 [50]. They showed, using clinically relevant dose fractionation protocols, that niraparib potently enhanced radiation-induced tumor growth delay in all four of these xenograft models. Niraparib, as a single agent, was previously shown to have potent antitumor activity in xenograft tumors made from the BRCA1 defective MDA-MB-436 cell line [27]. The results of the first phase I clinical trial of niraparib have been published recently showing that it is well tolerated and has antitumor activity in carriers of BRCA mutations and in patients with other cancers including NSCLC and prostate cancer [51]. The results of this trial and others have sparked a resurgence of interest in PARPi [52].

In conclusion, we have shown that the PARPi, niraparib, potently radiosensitizes human tumor cells derived from lung, breast and prostate cancers in a p53-independent manner. The mechanism to explain this sensitization appears to involve the conversion of radiation-induced, sublethal SSBs into lethal DSBs through inhibition of BER. Coupled with the previously reported in vivo activity and results from the phase I clinical trial, the present findings support the continued clinical assessment of niraparib in combination with DNA damaging agents including radiation.

## MATERIALS AND METHODS

### Cell cultures and reagents

The human cell lines A549, H1299, Calu-6, H460, CCD-16, MDA-MB-231, MDA-MB-436, MCF-10A and LnCaP were all obtained from the American Type Culture Collection (ATCC) and routinely maintained in RPMI-1640 medium supplemented with 10% fetal bovine serum (FBS), 10,000 U/ML of penicillin-streptomycin, and 2 mmol/L-glutamine. The identities of these cell lines were validated during the course of this study by short tandem repeat (STR) profiling conducted by the institution's Characterized Cell Line Core using the AmpFlSTR Identifiler PCR amplification kit according to the manufacturer's instructions (Applied Biosystems). The STR profiles for these cell lines matched their known ATCC fingerprints. Niraparib was provided by Merck Sharp & Dohme Corp., and its chemical structure has been described previously [27].

### Antibodies

The antibody to RAD51 (R1528) was purchased from Sigma, and the antibody to γ-H2AX (Ser139) clone JBW301 (05-636) antibody was purchased from Millipore.

### Clonogenic assay

The effectiveness of the combination of niraparib and ionizing radiation was assessed by clonogenic assays. Briefly, cells growing in log phase were treated with 1 μmol/L niraparib 1 h prior to irradiation. Following irradiation, the cells were subjected to a 24-h post-irradiation treatment with 1 μmol/L niraparib. The cells were then trypsinized and counted, and known numbers were seeded in 60-mm culture dishes in two sets of three for each dose of radiation. Sufficient numbers were seeded to ensure that about 30-100 macroscopic colonies would appear in each plate after 10-14 days. Colonies were stained with 0.5% gentian violet in methanol and counted. The plating efficiency (PE) for each dose was calculated by dividing the number of colonies by the number of cells plated and expressing the result as a percentage. The surviving fraction was calculated by dividing the PE of the treatment by the PE of the appropriate un-irradiated control.

### Immunofluorescence

A549 or H1299 cells were cultivated on coverslips placed in 35-mm dishes, irradiated with 2 Gy, and treated with 1 μmol/L niraparib as indicated. The medium was then aspirated, and the cells were rinsed briefly in PBS and then fixed with 2% paraformaldehyde for 15 min. Permeabilization was achieved by a 10-min incubation with 100% methanol at −20°C. After three 5-min rinses in PBS, the cells were incubated in blocking buffer (1X PBS, 50 μL/mL normal goat serum, and 0.3% Triton X-100) for 1 h at room temperature. Next, the cells were incubated in γ-H2AX (Millipore) or RAD51 (Sigma) primary antibody in antibody dilution buffer (1X PBS, 10 mg/mL bovine serum albumin, 0.3% Triton X-100) overnight at 4°C with gentle shaking. After being washed with PBS, primary antibodies were visualized after a 2-h incubation with the appropriate Alexa Fluor-conjugated secondary antibody (goat anti-rabbit FITC or goat anti-mouse Alexa Fluor 594) at a 1:500 dilution. Nuclei were counterstained with 1:500 4'6-diamidino-2-phenylindole dihydrochloride (DAPI) in PBS, and the coverslips were mounted on slides with Vectashield (Vector Laboratories). Slides were examined using a Leica fluorescence microscope equipped with a CCD camera and images were imported into Advanced Spot Image software. To quantify γ-H2AX or RAD51 foci, 50-100 nuclei were evaluated.

### PARP Assay

The inhibition of PARP was analyzed in A549 and H1299 cells using the HT Universal Chemiluminescent PARP Assay Kit (Trevigen) according to the manufacturer's instructions. Briefly, cells were treated with DMSO or 1 μmol/L niraparib for 15, 30, 60, or 120 minutes, trypsinized, and transferred to a pre-chilled tube. The cells were washed twice with ice cold PBS and resuspended in cold PARP extraction buffer. The cell suspensions were incubated on ice for 30 minutes with periodic vortexing to disrupt the cell membrane. The suspensions were centrifuged and the supernatant transferred to a pre-chilled tube on ice. The histone coated wells of the 96-well plate were rehydrated with 1X PARP buffer and incubated at room temperature for 30 minutes. The PARP buffer was removed and 20 μg of protein as determined by the Bio-Rad Protein Assay was added to each well followed by diluted PARP-HSA enzyme and 1X PARP buffer. The strip wells were then incubated at room temperature for 60 minutes, washed twice with PBS containing 0.1% Triton X-100, and then washed with PBS. Diluted Strep-HRP was added to the strip wells and incubated for 60 minutes at room temperature. The wells were washed again as before. Equal volumes of PeroxyGlow A and B were combined and added to the wells and chemiluminescent readings were obtained immediately using a plate-reader.

### Statistical Analysis

Statistical significance was assessed by t test (two sample assuming unequal variances) and expressed as mean ± standard error. A difference was considered significant if p<0.05.

## SUPPLEMENTARY FIGURE


