# Supplementary material for: Niraparib (MK-4827), a novel poly(ADP-Ribose) polymerase inhibitor, radiosensitizes human lung and breast cancer cells
Source: Oncotarget. 2014 Jun 9;5(13):5076–86. doi: 10.18632/oncotarget.2083 (PMC4148123; doi:10.18632/oncotarget.2083)
Supplement: Supplementary file 1 [file oncotarget-05-5076-s001.pdf]

Niraparib (MK-4827), a novel poly(ADP-Ribose) polymerase inhibitor, radiosensitizes human lung and breast cancer cells

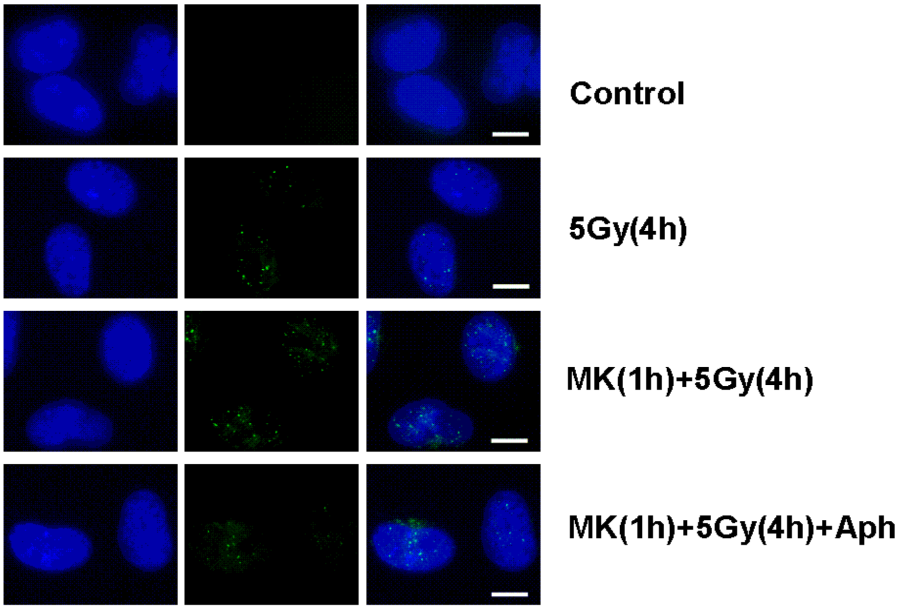

Figure S1: Representative photomicrographs of RAD51 foci from Figure 4D. Bar is 10 microns.
